# Anticipatory threat responses mediate the relationship between mindfulness and anxiety: A cross-sectional study

**DOI:** 10.3389/fpubh.2022.988577

**Published:** 2022-09-26

**Authors:** Yuanyuan Xu, Wenqiang Huang, Xiaofan Yan, Fang Lu, Min Li

**Affiliations:** ^1^Department of Military Psychology, Army Medical University, Chongqing, China; ^2^Department of Sleepy Psychosome, Chongqing Jiangbei Hospital of Traditional Chinese Medicine, Chongqing, China; ^3^Department of Nursing, Army Medical University, Chongqing, China

**Keywords:** anxiety, mindfulness, anticipatory threaten responses, intolerance of uncertainty, LPP

## Abstract

Increasing research has shown that mindfulness-based interventions can effectively alleviate anxiety; however, the underlying neural mechanism has not yet been elucidated. Recent studies suggest that abnormal and excessive anticipatory responses to unpredictable threats play an important role in anxiety symptoms. Mindfulness refers to the non-judgmental awareness of the present moment's real experience, which is antithetical to the future-oriented thinking processes involved in anxiety-oriented cognition and its corresponding emotion regulation tactics. Thus, mitigating anticipatory threat responses may be a potential mechanism by which mindfulness alleviates anxiety. This study aimed to detect the possible mediating effects of anticipatory threat responses on the relationship between mindfulness and anxiety. A total of 35 trait-anxious (TA) individuals and 36 low-anxious (LA) individuals were recruited to participate in the predictable and unpredictable threat test. Self-reported intolerance of uncertainty (IU) and electroencephalographic responses to uncertainty were recorded. TA individuals reported more IU and less mindfulness, and exhibited significantly higher late positive potential (LPP) and longer reaction time (RT) than LA individuals in the unpredictable negative threat condition. In addition, there were significant mediating effects of the LPP amplitude and RT in the uncertain threats on the relationship between mindfulness and anxiety. The data from this study verified that mitigating anticipatory threat responses (including self-reported IU, behavioral RT, and LPP amplitude) might be the potential mechanism by which mindfulness alleviates anxiety. These findings may have practical implications for the development and optimization of mindfulness treatments for anxiety.

## Introduction

Anxiety is a salient feeling of worry, nervousness, or unease when facing a threatening situation. People with pathological anxiety experience hypervigilance and increased behavioral responsivity in the absence of rationally fearful stimuli (1). A lifetime prevalence estimate of 28.8% places anxiety disorders as the most common class of mental disorders, causing serious damage to patients' social functioning (2). Furthermore, the COVID-19 pandemic has led to an 11.2% increase (95% uncertainty interval: 5.3–17.3) in cases of anxiety disorders in China in 2020 (3, 4), which heavily burdens both families and society.

Mindfulness-based interventions (MBIs) are a promising category of anxiety treatment. Mindfulness is the basic attentional stance underlying various Buddhist traditions, such as Theravada, Vajrayana and Mahayana (Zen), and has been called “the heart” (or “dharma” in Sansfrit) of Buddhist meditation, which was historically developed as a method to suspend personal suffering (5). Since it spread to the West, it has been increasingly applied in clinical settings. Mindfulness is commonly defined as the perception and acceptance of constantly changing experiences (6), which may include thoughts, emotions, somatic sensations, and responses to the external stimuli (7, 8). Evidence of its validity in alleviating anxiety comes from studies demonstrating negative relationships between mindfulness and anxiety (9, 10), intervention research (11–14), and meta-analysis (15–17). Over the past several decades, MBIs have been increasingly utilized among groups of relatively healthy individuals to promote wellbeing, as well as in a wide variety of clinical disorders, as a complement to cognitive or behavioral techniques to relieve mental distress (18, 19). The most common and well-studied MBIs are mindfulness-based cognitive therapy (MBCT) and mindfulness-based stress reduction (MBSR). MBSR is a manualized treatment program widely used to reduce psychological morbidity associated with chronic illnesses and treat emotional and behavioral disorders (6). MBCT is similar to and involves MBSR but emphasizes the ability to self-manage, control, and improve (17). Although empirical research on the effectiveness of mindfulness is increasing, the mechanisms through which mindfulness improves anxiety have rarely been investigated. One potential mechanism is an anticipatory response to uncertain threats (20).

Immoderate reactions to uncertain stimuli have been regarded as an intolerance of uncertainty (IU) (20). People with high IU are inclined to consider ambiguity a threat and are prone to overestimate the likelihood of an uncertain event and the cost of responding, thus resulting in maladaptive behaviors such as vigilance (i.e., paying more early phasic and sustained attention to uncertain target cueing) (21) and avoidance, which aims to decrease uncertainty (22). Recent perspectives consider anxiety to be a future-oriented emotional state; abnormal and excessive anticipatory responses under unpredictable threats explain the unique variance in anxiety psychopathology that contributes to stress and anxious behaviors (20, 23). Consistent with this perspective, a meta-analysis showed that IU is strongly associated with a range of symptoms in disorders, such as obsessive-compulsive disorder, panic disorder, social anxiety disorder, and agoraphobia, thus validating IU's trans-diagnostic importance (24).

Unlike the excessive anticipation reaction to potential future threats experienced in a state of anxiety, mindfulness refers to the non-judgmental awareness of the present moment's real experience, which is contrary to the future-oriented thinking involved in anxiety-driven cognition and its relevant emotion regulation strategies (7, 25). The anxiety-attenuating effects of MBIs have already been observed in the anticipatory phase for negative emotions, cortisol, and the autonomic nervous system (26), especially in the parasympathetic nervous system (27). Kim's research on panic disorders claimed that there was a significant correlation between the reduction in IU and relief of panic symptoms after MBCT (12). In line with this research, another cross-sectional study indicated that the benefits of mindfulness on anxiety symptoms are mediated by self-reported IU. However, this mediating effect was not confirmed in physiological responses (i.e., the startle reflex) to uncertain threats (20). One possible interpretation of this finding is that the IU questionnaire measured a higher-order cognitive process response to uncertainty while the startle reflex measured a lower-order defensive response to uncertainty.

Recent electrophysiological (EEG) studies have focused on the modulation of anxiety over threat anticipation by the intensity of uncertainty and found that, compared with certain cues, uncertain aversive cues elicit significantly larger stimulus-preceding negativity (SPN), P2 (a positive posterior deflection peaking at 200–250 ms), and late positive potential (LPP) (21, 28). The LPP is a centroparietal slow wave that seems to be modulated by higher-level cognitive processing (29) and has been demonstrated to be sensitive to stimulus predictability (30). With respect to uncertainty processing, studies have reported increased LPP amplitude for uncertain aversive cues compared with certain safe cues in threat-of-shock designs (21, 31). According to Grupe's “uncertainty and anticipation model of anxiety” (UAMA) theory (23), the overestimation of threat costs and probabilities causes exaggerated emotional and behavioral reactivity in anxious individuals. Thus, we chose LPP as an indicator of sustained cognitive processing in our research, which explores the potential mediating effects of uncertain threat responses on the association between mindfulness and anxiety and further investigates whether the relationships would vary according to the degree of threat exhibited by the stimuli (32).

Ongoing research has shown that the neural correlates of trait anxiety can predict pathological anxiety-driven behaviors (32). Thus, the present study focused on highly anxious individuals to explore the underlying mechanisms through which mindfulness alleviates anxiety. It was hypothesized that (1) both trait-anxious (TA) individuals and low-anxious (LA) individuals would express excessive anticipatory responses (including self-reported IU, behavioral reaction time (RT) and EEG responses) under conditions of threat uncertainty, and that the TA group's response would be more intense; (2) mindfulness would be negatively correlated with anxiety and IU; and (3) reactions to uncertainty might be the possible mechanism by which mindfulness alleviates anxiety.

## Materials and methods

### Participants

To recruit individuals with different levels of anxiety, recruitment advertisements were posted at the Army Medical University and three affiliated hospitals. Volunteers who scanned the quick response (QR) codes on the advertisements to sign up for the study were asked to complete the screening questionnaires (*n* = 191). The presence of psychiatric and neurological diseases was assessed through this screening questionnaires. We applied the following inclusion criteria: (1) aged 18–45 years, (2) right-handed individuals, (3) no history of neurological or psychiatric diagnosis, and (4) normal visual acuity or corrected visual acuity. In total, 162 subjects completed all questionnaire items and met all inclusion criteria. Based on their scores on the Trait Anxiety Inventory (TAI) in the State-Trait Anxiety Inventory (33), participants who scored 44 and above were classified as TA individuals while those with a score of 34 and below were classified as LA individuals (34). Thus, we invited 35 TA individuals and 36 LA individuals to participate in further EEG research. The intentions and project procedure of this study were then provided to qualified participants, and their written informed consent was obtained. This study was conducted in accordance with the Declaration of Helsinki. The regional ethics committee of the Army Medical University approved this consent procedure and the study protocol (reference number: 2020-019-02). Participants were paid 50 RMB after they finished this study.

**Figure 1:**
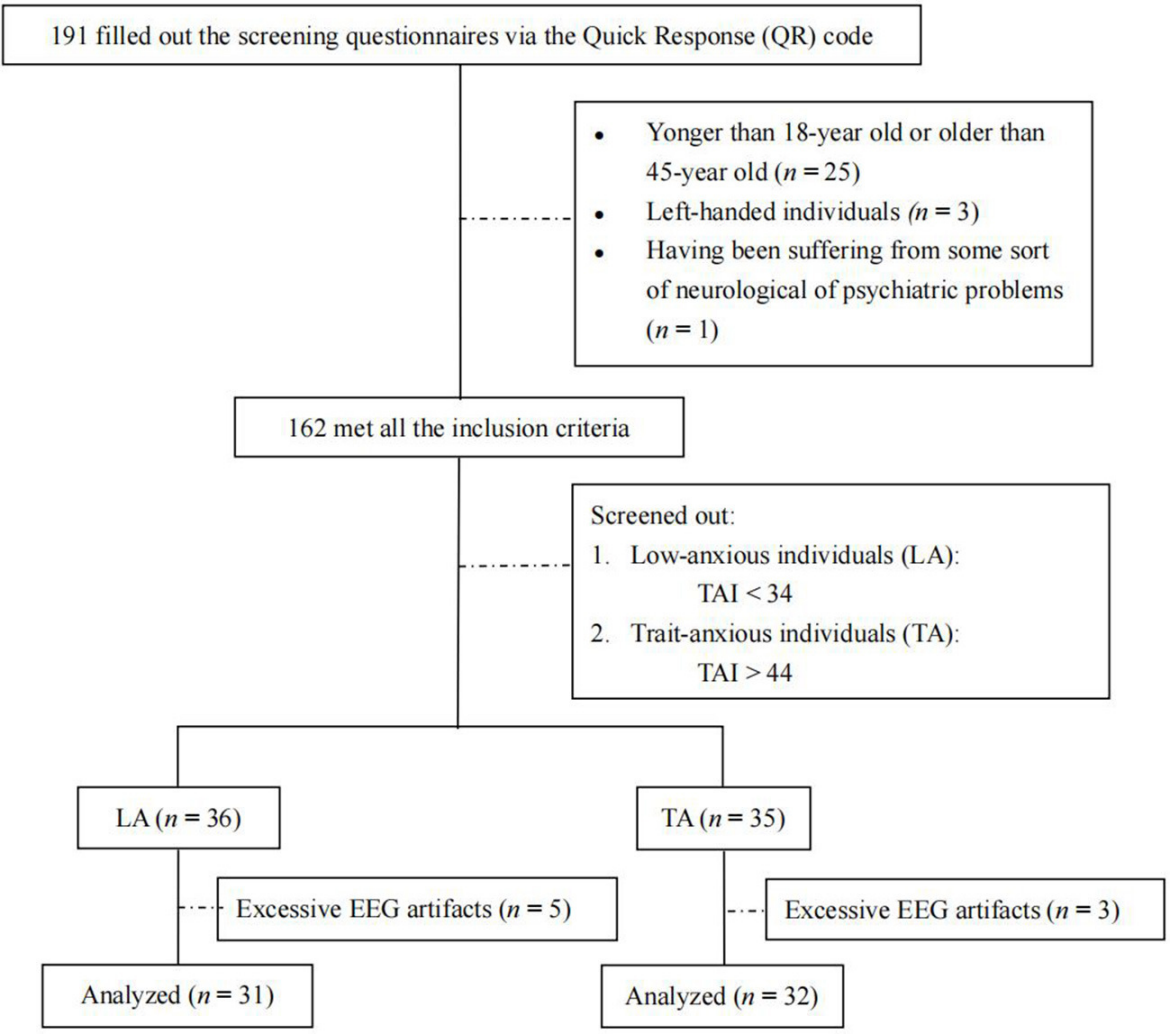
Flow of participants.

Figure 1 shows the participants' flowchart. A total of 71 participants (TA group = 35, LA group = 36) were eligible to participate in the EEG experiments. Eight subjects were excluded because of excessive EEG artifacts. The final sample comprised 63 participants (TA group = 32, LA group = 31). G^*^Power 3.1.9.7. was used to determine the sample size. Based on the input parameters specifying a medium effect size of f = 0.25, α = 0.05, 1-β = 0.95, number of groups = 2, and number of measurements = 4, we obtained a total sample size of N = 36. Thus, the sample size of our study (*n* = 63) was sufficient for statistical analysis.

### Task design

**Figure 2:**
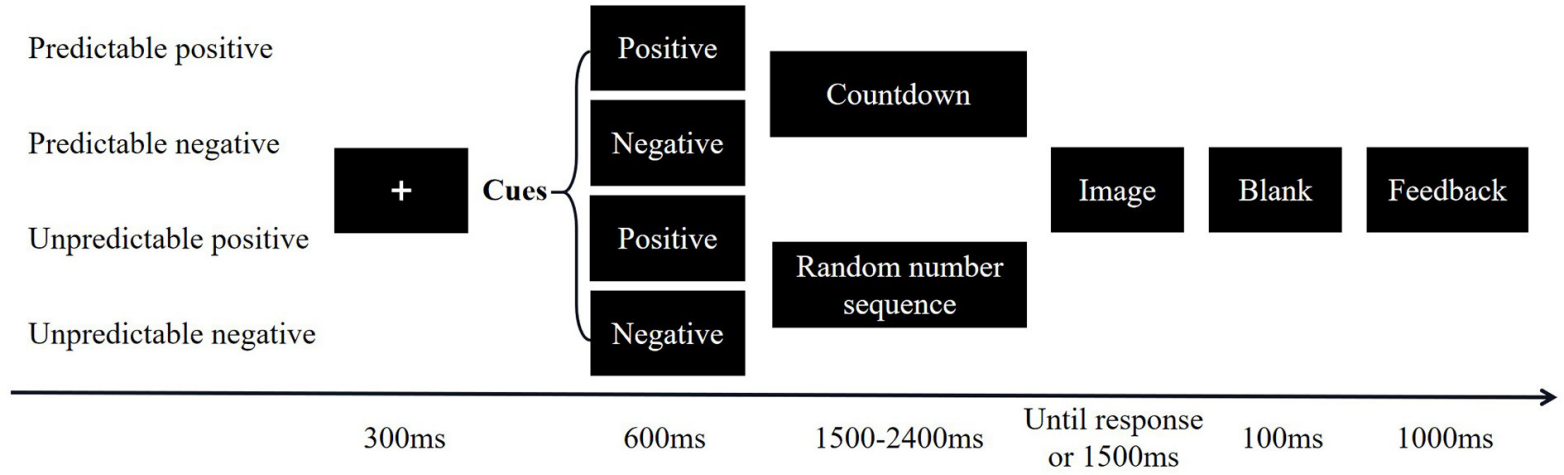
Schematic of experimental procedure.

Participants completed self-report questionnaires and EEG assessments under predictable and unpredictable conditions. The EEG recordings were conducted in a sound-attenuated room. The task was a modified version of a published threat test (35) that included four conditions: (1) predictable positive events (PP), (2) predictable negative events (PN), (3) unpredictable positive events (UP), and (4) unpredictable negative events (UN). Each condition contained 80 trials, with each trial consisting of a 300-ms fixation point presented at the center of the computer screen, followed by a 600-ms cue (i.e., “positive” or “negative”), after which a sequence of numbers would appear. Participants were told that a pleasant or aversive image (their valence was in accordance with the cues) would be shown on the screen after the sequence of numbers. During predictable conditions, the numbers would count down from any number between 10 and 6 to 1, at which point an image would appear. Under unpredictable conditions, the sequence of numbers would appear randomly, and pleasant or aversive images would be presented at any time. Pictures were presented for 1,500 ms and subjects were required to determine whether the scenes in the image occurred indoors (press “F” on the keyboard) or outdoors (press “J” on the keyboard). The image disappeared after the keystroke. After a 100-ms blank screen, feedback (correct or false) for participants' responses appeared (see Figure 2). The RT of the images was collected only for the correct response and then averaged for each condition.

The task consisted of two predictable runs and two unpredictable runs with a counterbalanced sequence. Each run consisted of 40 positive and 40 negative trials, and the order of the images was randomized. At the end of each run, a mandatory 30-s rest was provided. Before the formal experiment began, eight practice trials were performed to familiarize the participants with the paradigm. To ensure that all participants received the same information, instructions on the experimental procedures were standardized and displayed on a computer screen before the practice stage. The same researcher answered all questions throughout the study process.

### Materials

A total of 320 images were selected from the native Chinese Affective Picture System (CAPS) (36). A 9-point scale ranging from 1 (negative/calm) to 9 (positive/arousal) was used to evaluate each picture's valence and arousal degree. In the present study, the mean valence and arousal ratings of the selected 160 positive images were 6.71 ± 0.37 and 5.76 ± 0.58, respectively. The selected 160 negative images had a mean valence and arousal rating of 2.84 ± 0.83 and 5.01 ± 0.55, respectively. The valence and arousal ratings of the 160 pictures following the predictable cue and the 160 pictures following the unpredictable cue were not significantly different [valence rating: *t*(318,1) = −1.5, *p* = 0.13; arousal rating: *t*(318,1) = −0.98, *p* = 0.33]. Both predictable and unpredictable trials contained half of the positive and half of the negative images. Differences in the valence and arousal ratings between the 80 negative and 80 positive pictures for predictable trials [valence rating: *t*(158,1) = 33.43, *p* < 0.01; arousal rating: *t*(158,1) = 11.86, *p* < 0.01] were similar to the corresponding differences in unpredictable trials [valence rating: *t*(158,1) = 66.98, *p* < 0.01; arousal rating: *t*(158,1) = 5.91, *p* < 0.01].

### Questionnaires

#### Hospital anxiety and depression scale (HADS)

The HADS was constructed using the 7-item anxiety subscale and the 7-item depression subscale. A 4-point Likert scale ranging from 0 (not at all) to 3 (almost all the time) was used to evaluate participants' emotional states in the preceding month. The total scores ranged from 0 to 21, with a critical value of 9 for each subscale (37). The internal consistency coefficients of the anxiety subscale and the depression subscale for our sample were 0.90 and 0.89, respectively.

#### Trait anxiety inventory (TAI)

The 20-item TAI was administered to measure participants' general feelings of anxiety using a 4-point Likert scale (1 = barely, 4 = almost always). The total scores range from 20 to 80, with higher scores indicating greater trait anxiety (33). The Cronbach's alpha for our sample was 0.95.

#### Intolerance of uncertainty scale (IUS)

The IUS includes 11 items that assess how people react to uncertain situations in their lives. Participants rated the items on a 5-point Likert scale ranging from 1= “not at all characteristic of me” to 5= “entirely characteristic of me”. The total scores ranged from 11 to 55, with higher scores indicating less tolerance to uncertainty (38). The Cronbach's alpha for our sample was 0.96.

#### Five facet mindfulness questionnaire (FFMQ)

The 20-item FFMQ was used to measure five facets of mindfulness: observing, describing, acting with awareness, non-judging to inner experience, and non-reacting to inner experience. Participants rated the items on a 5-point Likert scale (1 = never or very rarely true, 5 = very often or always true) (39). The Cronbach's alpha for our sample was 0.84.

### EEG recording and data reduction

Continuous EEG data were collected using a 64 channel NuAmp acquisition system (Neuroscan Inc.) according to the international 10–20 system, with a reference at Cz and the ground placed between Fz and Fpz. Horizontal and vertical EEG activity was recorded from positions next to the outer rims of each eye and from above and below the right eye, respectively. The sampling rate was 1,000 Hz, and the impedances of all electrodes were below 5kΩ.

Offline, a digital average mastoid reference, (M1+M2)/2, was performed. The raw EEG data were bandpass filtered from 0.01 Hz to 30 Hz and manually scored for muscle and eye movement artifacts. They were segmented from 100 ms before cue onset to 2,000 ms afterward, referred to as baseline −100 to 0 ms before cue onset. Six electrodes–CP3, CPz, CP4, P3, Pz, and P4–were selected for further analysis, since the LPP in existing research was detected mostly in the centro-parietal region of the scalp (21). The inspection of the EEG data revealed a late positive component (with an onset of approximately 400 ms and an offset of approximately 1,100 ms) over parietal occipital sites. The mean amplitude of the LPP was extracted for further analysis (see Supplementary Table 1).

### Data analyses

Data analyses were performed using SPSS 19.0 and AMOS 20.0. The Shapiro-Wilk normality test was used to assess data or variable distribution. Age, HADS_A, TAI, and IUS did not show a normal distribution; therefore, comparisons were made by the Mann-Whitney *U* test. The differences in demographic variables (except Age) and FFMQ between TA group and LA group were analyzed by independent samples *t-tests* and chi-square tests. After splitting the data by group, a 2 (Groups: TA, LA) × 4 (Conditions: PP, UP, PN, and UN) repeated-measures analysis of variance (rmANOVA) was applied to examine the discrepancies in behavioral RT and LPP amplitude with respect to the four stimuli conditions for the TA and LA groups. Greenhouse–Geisser correction was applied to correct all ANOVA results. The Sidak correction was used to correct alpha for multiple comparisons. The Spearman rank-order correlation was used to calculate the relationships between variables. The *cocor* was used to conduct statistical comparisons between correlations (40). The mediation hypothesis was tested with structural equation modeling (SEM). Indices including CMIN/DF (a value between 1 and 3 indicates acceptable fit between the hypothetical model and the sample data), adjusted goodness-of-fit index (AGFI, a value >0.90 indicates acceptable model fit) (41), and root-mean-square error of approximation (RMSEA, a value between 0.05 and 0.08 reflects reasonable model fit) (42) were calculated to assess the overall model fit.

## Results

### Self-report measures

According to the parametric and non-parametric test results, group differences in the demographic variables were not significant (see Table 1). Table 2 shows the means and standard deviations for all self-reported variables for the TA and LA groups. According to the independent samples *t*-test and Mann-Whitney U test, the grouping effects for all self-reported variables were significant, with lower scores for FFMQ and subscales (except for the observing subscale), and higher scores for HADS-A, TAI, and IUS in the TA group. The results indicated that the participants in the TA group were more intolerant of uncertainty, tended to feel more stress, and had less mindfulness than emotionally healthy participants.

**Table 1:** Between-group differences regarding demographic data.

	**LA**, ***n*** = **31**		**TA**, ***n*** = **32**		**Total**, ***n*** = **63**	
	**Mean**	***SD***	**Mean**	***SD***	**Mean**	***SD***
**Age**	21.45	3.54	22.63	6.66	22.05	5.35
	***n***	**%**	***n***	**%**	***n***	**%**
**Gender**						
Male	24	77.42	27	84.38	51	80.95
Female	7	22.58	5	15.62	12	19.05
**Educational background**						
Junior high school diploma	0	0	3	9	3	4.76
Senior high school diploma	4	12.90	5	16	9	14.29
College degree	24	77.42	24	75	48	76.19
Graduate degree	3	0	0	0	3	4.76
**Marital status**						
Single	30	96.77	28	87.50	58	92.06
Married	1	3.23	4	12.50	5	7.94

Using Mann-Whitney U tests and Chi-square tests, between-group differences in the demographic variables were not statistically significant. LA, Low-anxious individuals; TA, Trait-anxious individuals.

**Table 2:** Scores on self-report scales for the LA (*n* = 31) and TA (*n* = 32).

	**LA *M (SD)***	**TA *M (SD)***	***p***
HADS-A	10.19 (1.92)	17.47 (3.11)	<0.001
TAI	29.26 (3.39)	54.94 (5.91)	<0.001
IUS	21.94 (8.70)	40.63 (6.98)	<0.001
FFMQ	71.61 (7.32)	56.91 (9.23)	<0.001
FFMQ_Observing	13.77 (3.30)	12.94 (2.75)	0.278
FFMQ_Describing	15.10 (3.03)	11.09 (2.75)	<0.001
FFMQ_Acting with awareness	16.16 (2.42)	10.78 (3.77)	<0.001
FFMQ_Non-judging to inner experience	13.35 (2.97)	11.00 (2.55)	0.002
FFMQ_Non-reacting to inner experience	13.35 (2.90)	11.09 (3.31)	0.005

LA, Low-anxious individuals; TA, Trait-anxious individuals; M, Means; SD, Standard Deviations; HADS-A, Anxiety subscale of Hospital Anxiety and Depression Scale; TAI, Trait Anxiety Inventory; FFMQ, Five Facet Mindfulness Questionnaire; IUS, Intolerance of Uncertainty Scale.

### RT to the images

Table 3 presents the results of the rmANOVA after splitting the RT to analyze the TA and LA groups across the four conditions. Although no significant group × condition interaction was found, there was a significant main effect of the condition. For all participants, the mean RT during the UN condition was significantly longer than those during the other three conditions, and the mean RT during PN was significantly longer than that during PP. The rmANOVA for RT also revealed a significant main effect of the group. The mean RT of TA group was significantly longer than that of the LA group, particularly with UN stimuli. Based on the results, we found that participants waited longer to press keys on uncertain and negative stimuli, especially in the TA group.

**Table 3:** Means (*M*), Standard Deviations (*SD*) and the results of rmANOVA for RT.

	***N***	**PP *M(SD)***	**UP *M(SD)***	**PN *M(SD)***	**UN *M(SD)***	**Within subject effect *F(df)***	**Interaction Effect *F(df)***	**Between subject effect *F(df)***
LA	31	646.13 (14.38)^a^^b^^c^	660.90 (14.46)^a^^b^	673.68 (15.31)^a^^b^	711.11 (16.63)^a^	54.51^***^ (2.49, 151.81)	0.44 (2.49, 151.81)	10.52^***^ (1, 61)
TA	32	712.04 (14.16)^b^^c^	718.36 (14.24)^b^	741.07 (15.07)^b^	780.53 (15.95)			

LA, Low-anxious individuals; TA, Trait-anxious individuals; UP, Unpredictable positive events; PP, Predictable positive events; PN, Predictable negative events; UN, Unpredictable negative events; RT, Reaction time; M, Means; SD, Standard Deviations.

*** *p* < 0.001.

a *p* < 0.05 for rmANOVA post hoc test for LA – TA.

b *p* < 0.0087 (Sidak correction) for rmANOVA post hoc test for UP/PP/PN – UN.

c *p* < 0.0087 (Sidak correction) for rmANOVA post hoc test for PP – PN.

### LPP

**Figure 3:**
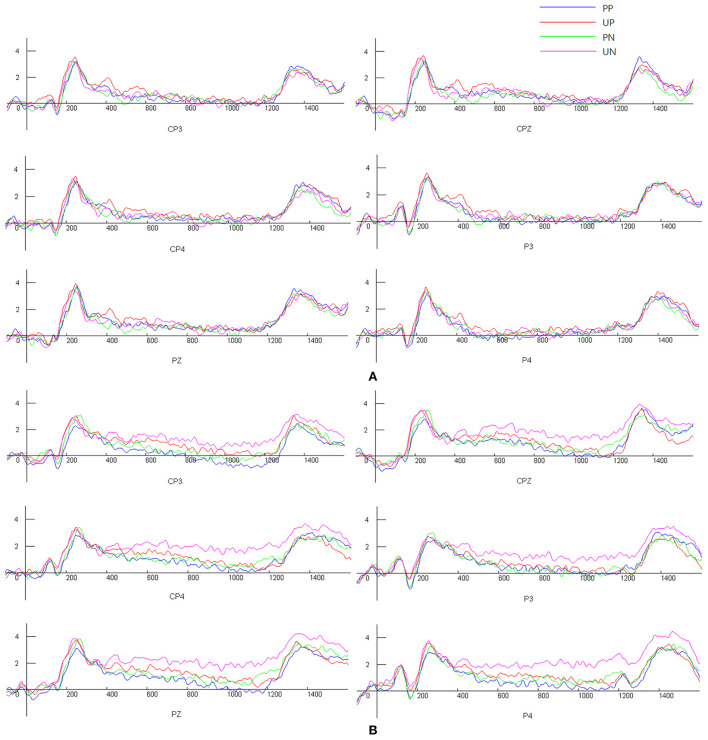
Participants' mean levels of LPP amplitude [**(A)** Low-anxious individuals, **(B)** Trait-anxious individuals]. UP, Unpredictable positive events; PP, Predictable positive events; PN, Predictable negative events; UN, Unpredictable negative events.

Table 4 presents the significant main effect of condition and group × condition interactions at the three occipital electrodes (i.e., CP3, PZ, and P4). Follow-up planned comparisons indicated that for the TA group, the amplitude of LPP during trials signaling unpredictable negative stimuli was greater than that of LPP during trials signaling certain positive stimuli (*p* < 0.009). Although there were upward trends from certain conditions to unpredictable conditions and from positive to negative stimuli on the amplitude of LPP for the LA group, the changes were not statistically significant. In addition, within the unpredictable negative condition, the TA group demonstrated significantly higher LPP than the LA group (*p* = 0.02, CP4 and 0.01, P4), whereas in the other three conditions (PP, UP, PN), the amplitude of LPP to cues did not differ significantly between groups (see Table 4, Figure 3).

**Table 4:** Results of the rmANOVA on two groups and four conditions for LPP.

		**PP**	**UP**	**PN**	**UN**	**Interaction effect**
		***M (SD)***	***M (SD)***	***M (SD)***	***M (SD)***	***F(df)***
CP3	LA	0.22 (0.35)	0.35 (0.39)	0.38 (0.44)	0.26 (0.40)	2.93^*^ (2.87, 175.29)
	TA	−0.21 (0.34)^a^	0.15 (0.38)	0.59 (0.43)	1.10 (0.40)	
CPZ	LA	0.49 (0.38)	0.45 (0.39)	0.66 (0.46)	0.54 (0.47)	2.22 (2.86, 174.67)
	TA	0.57 (0.37)^a^	0.78 (0.39)	0.95 (0.46)	1.75 (0.46)	
CP4	LA	0.37 (0.35)	0.32 (0.39)	0.55 (0.45)	0.33 (0.45)	2.79 (2.72, 165.94)
	TA	0.60 (0.34)^a^	0.82 (0.38)	1.01 (0.44)	1.91 (0.45)^b^	
P3	LA	0.14 (0.33)	0.28 (0.41)	0.37 (0.40)	0.32 (0.35)	2.26 (2.67, 162.99)
	TA	0.25 (0.32)^a^	0.21 (0.40)	0.47 (0.40)	1.26 (0.34)	
PZ	LA	0.66 (0.34)	0.66 (0.39)	0.72 (0.43)	0.77 (0.46)	3.57^*^ (2.75, 167.47)
	TA	0.44 (0.34)^a^	0.86 (0.38)	1.09 (0.42)	2.04 (0.45)	
P4	LA	0.18 (0.33)	0.31 (0.43)	0.43 (0.42)	0.31 (0.43)	3.23^*^ (2.71, 165.25)
	TA	0.44 (0.33)^a^	0.85 (0.42)	0.97 (0.41)	2.03 (0.42)^b^	

LPP, late positive potential; LA, Low-anxious individuals; TA, Trait-anxious individuals; UP, Unpredictable positive events; PP, Predictable positive events; PN, Predictable negative events; UN, Unpredictable negative events; CP3; CPz; CP4; P3; Pz and P4, electrodes on the centro-parietal region of the scalp.

* *p* < 0.05.

a *p* < 0.0087 (Sidak correction) for rmANOVA post hoc test for PP – UN.

b *p* < 0.05 for rmANOVA post hoc test for LA-TA. The bold values indicates statistically significant *p*-values.

### Mediation model of anticipatory response to uncertain threats on mindfulness improving anxiety

The intercorrelations between all variables are summarized in Table 5. IUS scores, RT, and LPP amplitude to uncertain threats were all significantly related to scores on measures of anxiety symptoms and were inversely related to FFMQ scores. These results support the supposed correlations and allow for further mediation analyses. Self-reported IUS was significantly associated with RT to the images in both anxiety groups. No significant relationship between IUS scores and LPP amplitude was found.

**Table 5:** Correlations between all variables.

	**1**	**2**	**3**	**4**	**5**	**6**	**7**	**8**	**9**	**10**	**11**
1. HADS_A	-										
2. FFMQ	−0.61^**^	-									
3. IUS	0.83^**^	−0.63^**^	-								
4. RT_UP	0.32^*^	−0.22	0.31^*^	-							
5. RT_PP	0.37^**^	−0.27^**^	0.35^**^	0.87^**^	-						
6. RT_PN	0.39^**^	−0.26^*^	0.34^**^	0.87^**^	0.94^**^	-					
7. RT_UN	0.44^*^	−0.25^*^	0.38^**^	0.88^**^	0.88^**^	0.90^**^	-				
8. LPP_PP	0.13	−0.14	−0.02	−0.09	−0.1	−0.09	−0.07	-			
9. LPP_UP	0.17	−0.09	−0.04	−0.01	−0.07	−0.07	−0.09	0.62^**^	-		
10. LPP_PN	0.18	−0.10	0.09	0.14	0.05	0.06	0.14	0.61^**^	0.51^**^	-	
11. LPP_UN	0.28^*^	−0.30^*^	0.17	−0.04	0.01	0.07	0.02	0.60^**^	0.48^**^	0.57^**^	-

HADS-A, Anxiety subscale of Hospital Anxiety and Depression Scale; IUS, Intolerance of Uncertainty Scale; FFMQ, Five Facet Mindfulness Questionnaire; RT, Reaction time; LPP, Late positive potential; UP, Unpredictable positive events; PP, Predictable positive events; PN, Predictable negative events; UN, Unpredictable negative events.

* *p* < 0.05;

** *p* < 0.01.

**Figure 4:**
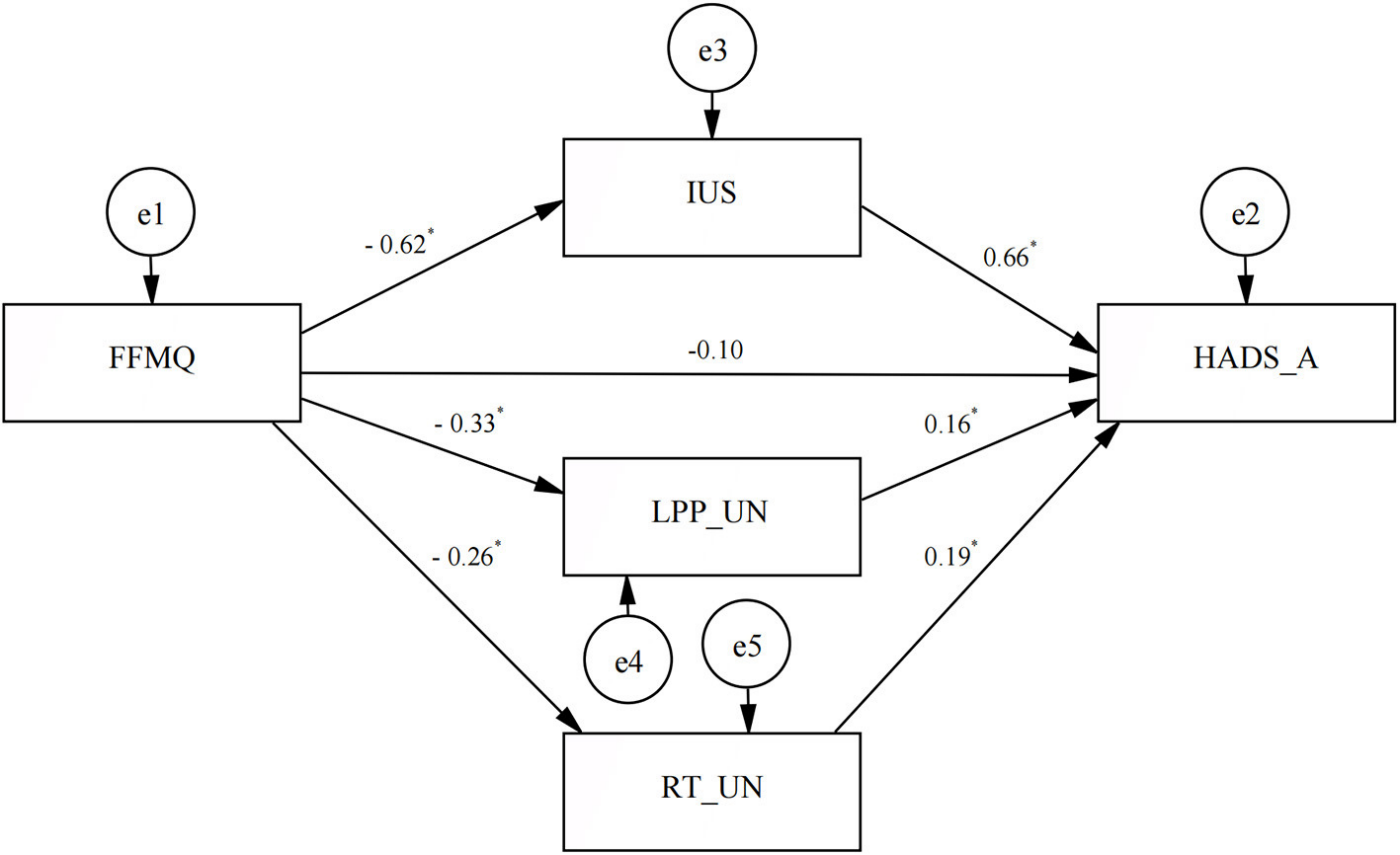
Mediation model of anticipatory response to uncertain threats to mindfulness improving anxiety. FFMQ, Five Facet Mindfulness Questionnaire; IUS, Intolerance of Uncertainty Scale; HADS-A, Anxiety subscale of Hospital Anxiety and Depression Scale; LPP-UN, the amplitude of LPP during trials signaling unpredictable negative stimuli; RT-UN, mean reaction time to the unpredictable negative stimuli. *p < 0.05.

The SEM results demonstrated that the overall model yielded a satisfactory fit, CMIN/DF = 1.68, RMSEA<0.08, and AGFI = 0.99. All specific indirect effects on anxiety *via* anticipatory responses to uncertain threats, including IUS scores, RT, and LPP amplitude to uncertain threats, were significant (all *p* < 0.05, see Figure 4). There was no significant direct effect of mindfulness on anxiety. However, anticipatory responses to uncertain threats were found to mediate the association between mindfulness and anxiety. The standardized indirect effects of IU scores, RT, and LPP amplitude on uncertain threats were −0.40, −0.05, and −0.05, respectively (all *p* < 0.05).

## Discussion

Uncertainty about future threats is disruptive in anxiety. The current research aimed to explore whether an excessive threat response (both self-reported IU and behavioral and EEG responses to uncertain threats) significantly mediates the negative relationship between mindfulness and anxiety and to further investigate whether the relationship would vary according to various degrees of anxiety.

### Excessive anticipatory response to uncertain threats

Substantial research has shown that excessive reactions to uncertainty play a crucial role in pathological anxiety (21, 23, 43). According to the study results, in comparison with LA individuals, there was greater self-reported IU in TA group, which supports the previous findings of a positive relationship between IU and anxiety (20). Longer RT before key presses in the uncertain negative stimuli was found in both groups, suggesting that individuals might be more involved in uncertain threats. Moreover, according to the attentional control theory, increased attention consumes limited cognitive resources and less impairs the performance efficiency of concurrent task processing (44, 45).

In addition, LPP was recorded while the subjects were exposed to predictable and unpredictable conditions. As hypothesized, the LPP amplitude of the TA group regarding uncertain threats was significantly higher than that of the LA group. Enhanced LPP during trials signals unpredictable threats, suggesting increased salience and sustained higher-level cognitive processing for these cues. Previous EEG research has investigated uncertainty-related dynamics in attentional allocation and sustained stimulus elaboration in a cued-picture paradigm. The results showed larger P2 and LPP amplitudes in uncertain conditions, suggesting that the threat uncertainty context could enhance individuals' ability for early attentional capture and late top-down allocation of attention to stimuli (21).

Further, the present study contrasted participants with high and low TA to demonstrate that this trend of excessive anticipatory response to uncertain threats is more pronounced in the TA group. It seems that people with higher TA may demonstrate lower cognitive flexibility. They tend to prioritize uncertain threats and experience more difficulty adapting to new information (46), which may predispose them to pathological anxiety-driven behaviors (32).

Correlation analysis showed that self-reported IUS was significantly associated with RT to the images in both anxiety groups, which is consistent with a previous research (47). However, the relationship between IUS and LPP amplitude was not statistically significant. The result was inconsistent with Nelson et al.'s research (48). In their study, participants were invited to complete a passive fear generalization paradigm, and the research found that prospective IU (IUS-P, and not inhibitory IUS) was negatively correlated with LPP amplitude in the face of uncertainty, suggesting that individuals high in IUS-P might engage in cognitive avoidance during the threat uncertainty condition. Nevertheless the present study used a different version of IUS and conducted a different task that required participants' feedback. Thus, future research should adopt an experimental paradigm that includes trials that (1) only need participants' passive observation and (2) need their active feedback to further investigate the relationship between IUS and LPP amplitude.

### Anticipatory threat responses mediating the benefits of mindfulness on anxiety symptoms

A previous cross-sectional study conducted by Kraemer et al. affirmed that self-reported IU mediates the relationship between mindfulness and health anxiety (10). However, research on the relationship between mindfulness, anxiety, and physiological responses (i.e., the startle reflex) in the unpredictable threat condition has shown mixed results (20). The authors explained that this might be due to the IU scale measuring a higher-order cognitive response to uncertainty involving cognitive processes such as attention, working memory, and metacognition (49) while the startle magnitude measures a lower-order defensive response to uncertainty (20), and the latter does not seem necessary for the conscious experience of any emotional cognitive state (50). Thus, in the current study, the LPP amplitude to uncertain negative stimuli was chosen as an indicator of higher-level cognitive processing (29). As we assumed, the results demonstrated significant mediating effects of excessive threat response (both self-reported IU as well as RT and LPP amplitudes to uncertain threats) on the beneficial effects of mindfulness on anxiety.

Over the past few decades, MBIs have become increasingly ideal therapeutic strategies for relieving anxiety (15, 16). Of the mindfulness elements, the non-judging awareness of the present moment's real experience was strongly associated with anxiety symptoms (7, 9). This mindful awareness of the present moment could allow anxious individuals to avoid future-oriented thinking and the overestimation of a threat's costs and related possibilities (23), thereby mitigating individuals' threat anticipation of an uncertain event from excessive expectations to more reasonable expectations and eventually to non-judgmental acceptance (23, 51). Moreover, mindful acceptance might help decrease the defensive motivation elicited by uncertainty and instead strengthen one's ability to allow an experience to be as it is, thereby relieving intolerance and inflated anxiety about potential threats (20, 21).

Smaller amplitudes of LPP during trials signaling unpredictable negative stimuli and shorter RT before key presses in uncertain threats were observed in people with higher degrees of mindfulness. It could be speculated that people with higher mindfulness would appear less blocked by uncertain threats and that under uncertainty, their cognitive resources would be more flexibly deployed according to circumstantial demands (21). Thus, higher mindfulness would contribute to alleviating negative reactions to unpredictable stimuli, in that uncertainty would be less likely to be identified as something that is unacceptable or needs to be stopped (20), ultimately relieving anxiety.

This study provides insight into mindfulness interventions for individuals with anxiety. Mindfulness practices would work well on higher-level cognitive processing by guiding anxious individuals to: (1) observe the present moment rather than worry about the future so they are less involved in the potential threat that they imagine might happen and (2) act with awareness and allow everything (including the thoughts in the mind) to just be as it is rather than trying to control them. This approach does not mean that there is no coping with mental distress, but that there is a way to respond consciously in a state of awareness without judgment (19), which could help individuals with anxiety reduce their automatic avoidance of pain since mental discomfort is often unavoidable and a failure to cope often brings more anxiety.

### Limitations and future research

First, the mediating effect of the anticipatory threat responses on the association between mindfulness and anxiety was based on a cross-sectional survey. Further intervention studies are warranted, and responders and non-responders should be compared to measure the causal nature of these relationships. Second, the findings were based on 63 individuals who were either emotionally healthy or trait anxious and did not include anyone with clinical anxiety disorders, which may undermine the significance of several relationships between variables. For example, the correlation between IU and LPP amplitude in response to uncertain threats was only marginally significant in the present study (*p* = 0.056). Third, we did not inquire about previous contemplative/meditative or body-mind practices. Such practices may affect the responses of the participants, entailing a risk of reporting bias. Given the role of social desirability in self-reported measures, the study itself may also entail a risk of self-reporting bias, which may have affected the selection of TA and LA respondents, as well as the response of the participants to other measures (e.g., IUS and HADS). In addition, the sample size was small, which limits the power of the study to detect possible relationships and mediating effects. Therefore, a large-scale intervention study involving adequately-powered samples with comparable experiences of contemplative/meditative or body-mind practices and heightened symptom levels on multiple anxiety dimensions is necessary to replicate these findings in future research.

## Contribution to the field

Anxiety disorders are associated with substantial functional impairment and imposes a heavy burden on both families and society. Many studies have shown that MBIs can effectively alleviate anxiety; however, the underlying neural mechanism has not yet been elucidated. Research based on self-reported IU suggests that alleviating higher-order cognitive responses to uncertainty might mediate the effect of mindfulness on anxiety symptoms. Accordingly, the current study collected the LPP amplitudes in response to uncertain negative stimuli as the physiological indicators of higher-level cognitive processing. The results demonstrated significant mediating effects of LPP amplitude and RT on uncertain threats in the relationship between mindfulness and anxiety. The results provide further evidence that reactions to uncertain threats may play a role in the association between mindfulness and anxiety and suggest that interventions are needed to specifically target excessive anticipatory responses to uncertain threats.

## Conclusion

In summary, the current research demonstrated that unpredictable, high-threat conditions might trigger a more intense anticipatory response (including self-reported IU, behavioral RT, and LPP amplitude) in TA. It further verified that mitigating anticipatory threat responses might be the potential mechanism by which mindfulness alleviates anxiety. These findings lay important groundwork for understanding the role of strong intolerance of potential threats in the development and maintenance of anxiety and may have practical implications for informing the development and optimization of mindfulness treatments for anxiety.

## Data availability statement

Data used in the present analysis are included in the article/Supplementary material, further inquiries can be directed to the corresponding author/s.

## Ethics statement

The studies involving human participants were reviewed and approved by the Ethics Committee of Army Medical University. The patients/participants provided their written informed consent to participate in this study.

## Author contributions

YX: designed and executed the study, assisted with the data analyses, gained ethical approval, and wrote the paper. WH: recruited the subjects, collaborated with the design, and writing of the study. XY: collaborated with the recruiting of the subjects and analyzed the data. FL: collaborated with material preparation and data collection. ML: collaborated in the writing and editing of the final manuscript. All author approved the final version of the manuscript for submission.

## Funding

This study was financially supported by National Natural Science Foundation of China (No. 31700958).

## Conflict of interest

The authors declare that the research was conducted in the absence of any commercial or financial relationships that could be construed as a potential conflict of interest.

## Publisher's note

All claims expressed in this article are solely those of the authors and do not necessarily represent those of their affiliated organizations, or those of the publisher, the editors and the reviewers. Any product that may be evaluated in this article, or claim that may be made by its manufacturer, is not guaranteed or endorsed by the publisher.
